# Butyric Acid Added Apically to Intestinal Caco-2 Cells Elevates Hepatic ApoA-I Transcription and Rescues Lower ApoA-I Expression in Inflamed HepG2 Cells Co-Cultured in the Basolateral Compartment

**DOI:** 10.3390/biom11010071

**Published:** 2021-01-07

**Authors:** Jehad Z. Tayyeb, Herman E. Popeijus, Ronald P. Mensink, Jogchum Plat

**Affiliations:** 1 Department of Nutrition and Movement Sciences, NUTRIM School for Nutrition and Translational Research in Metabolism, Maastricht University, 6229 ET Maastricht, The Netherlands; j.tayyeb@maastrichtuniversity.nl (J.Z.T.); r.mensink@maastrichtuniversity.nl (R.P.M.); j.plat@maastrichtuniversity.nl (J.P.); 2 Department of Clinical Biochemistry, Faculty of Medicine, University of Jeddah, 23218 Jeddah, Saudi Arabia

**Keywords:** ApoA-I, transwell, NF-κB, mRNA, SCFAs

## Abstract

Apolipoprotein A-I (ApoA-I) concentrations are decreased during inflammation, which may reduce high-density lipoprotein (HDL) functionality. Thus, rescuing ApoA-I concentrations during inflammation might help to prevent atherosclerosis. Recent studies have shown that butyric acid (C4) has anti-inflammatory effects and rescues ApoA-I production. However, whether intestinal short chain fatty acids (SCFAs) are able to influence hepatic processes is unknown. Therefore, we investigated C4 anti-inflammatory effects on ApoA-I transcription in the intestine-liver co-culture model. C4 dose-response experiments in the presence or absence of cytokines were performed in a co-culture system including Caco-2 cells, HepG2 cells, or both. Changes in ApoA-I transcription in Caco-2 cells and HepG2 cells were analyzed using qPCR. C4 increased ApoA-I expression in HepG2 cells that cultured alone. When both cells were cultured together, C4 decreased ApoA-I expression in Caco-2 cells and increased ApoA-I expression in HepG2 cells. However, adding C4 to apical Caco-2 cells resulted in a smaller effect in HepG2 cells compared with adding C4 directly to the hepatocytes. Moreover, C4 rescued ApoA-I expression in inflamed HepG2 cells. These findings suggests that intestinal SCFAs can affect hepatic processes. However, the smaller effect in the co-culture experiment indicates cross-talk between intestine and liver.

## 1. Introduction

A higher production of short chain fatty acids (SCFAs) in the intestinal lumen may result in various health benefits [1]. Butyric acid (C4), the most studied SCFA, is mainly produced by colonic microbiota through the fermentation of water-soluble dietary fibers like pectin [2]. In the past, it has been shown that C4 plays a local role in the physiology of the intestinal mucosa, since amongst other functions it is the major oxidative substrate for colonocytes [3]. In addition, more recent studies have shown that C4 has local anti-inflammatory effects in human colonic epithelial cells [4]. In fact, C4 was reduced in intestinal biopsies of Crohn’s disease patients’ mRNA expression and production of pro-inflammatory cytokines, such as TNFα, by the inhibition of nuclear factor kappa B (NF-κB) transactivation [5]. Beside these local benefits, SCFAs also exert systemically beneficial effects, such as lowering inflammation in macrophages and endothelial cells through the inhibition of inflammatory cytokine production [6] and abating the development of atherosclerosis [7]. These latter anti-atherosclerotic effects are thought to be mediated by modulating pro-inflammatory cytokine production, endothelial dysfunction and oxidative stress [8]. Additionally, we have recently postulated that SCFAs may also affect atherosclerotic risk via elevating hepatic Apolipoprotein A-I (ApoA-I) transcription [9]. To be effective systemically, SCFAs first need to be absorbed from the intestinal lumen by the enterocytes and transported into the circulation via the portal vein [10]. This makes it tempting to suggest that SCFAs will most likely affect hepatic (patho)physiology, as shown, for example, in previous studies using liver (HepG2) cells [9,11]. In particular, the observation that C4 could rescue the inflammation-induced reduction in ApoA-I transcription [12] is highly relevant, since ApoA-I is a negative acute phase protein, and its production is significantly lowered during inflammation [13]. This consequent reduction in serum ApoA-I concentrations seems undesirable, especially during inflammation when the numerous beneficial effects of the ApoA-I protein itself [14,15] are needed. However, in our earlier experiments mimicking hepatic inflammation, C4 was added directly to the medium of the HepG2 cells, while as mentioned earlier, SCFAs are formed in vivo in the intestinal lumen and need to be transported to the liver before any hepatic effects can be expected. However, various SCFAs do not reach the liver in similar concentrations since amounts produced and oxidation within the enterocytes might differ [16]. To the best of our knowledge, the effects of butyrate on ApoA-I gene expression in the intestinal cells and the possible existence of cross-talk between enterocytes and hepatocytes on ApoA-I transcription are unknown. Theoretically, intraluminal-produced butyrate that is taken up by enterocytes might have a direct effect on hepatocytes when it reaches the liver [17]. However, butyrate may also change the secretion of certain factors by intestinal cells [18], which affect the transcription of ApoA-I in liver cells. Therefore, to understand this in more detail, we here investigated changes in ApoA-I transcription after adding C4 to Caco-2 cells or HepG2 cells directly and compared these changes to the effects of adding C4 to the apical surface of Caco-2 cells, while analyzing ApoA-I expression in HepG2 cells cultured in the basolateral compartment in our co-culture model. These co-culture studies were performed in normal as well as under inflamed conditions.

## 2. Material and Methods

### 2.1. Materials

Human epithelial colorectal adenocarcinoma (Caco-2) cells were obtained from ATCC (Molsheim, France). Human hepatocellular liver carcinoma cells (HepG2) were kindly provided by Sten Braesch-Andersen (Mabtech, Nacka Strand, Sweden). Cell culture flasks, plates and polyester membrane inserts (12 mm diameter, 0.4 µm pore diameter) were obtained from Corning (Cambridge, MA, USA). Dulbecco’s modified eagle medium (DMEM), Minimum essential medium (MEM), and sodium pyruvate and non-essential amino acids (NEAA) were all obtained from Thermo Fisher Scientific (Bleiswijk, The Netherlands). Fetal bovine serum (FBS) was purchased from PAA (Toronto, ON, Canada). Butyric acid (C4) was bought from Sigma (Uithoorn, The Netherlands). The BET inhibitor JQ1(+) was obtained from Bio-Techne—R&D (Minneapolis, MN, USA). Tumor necrosis factor-alpha (TNFα), interleukin-1beta (IL-1β), dimethyl sulfoxide (DMSO), and Tri-reagent were all purchased from Sigma (Uithoorn, The Netherlands).

### 2.2. Cell Culture and C4 Treatment

**Figure 1:**
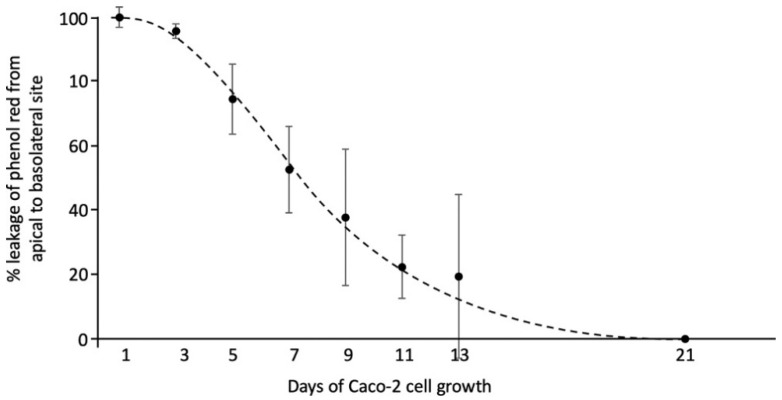
The percentage of phenol red leakage from the apical to the basolateral compartment in the transwell system. Medium solution (MEM) with phenol red was added to the apical compartment, while MEM without phenol red was added to the basolateral compartment. The optical density (OD) of the medium solution in both compartments was measured across the different days of Caco-2 cell growth. All results are presented as the mean, while error bars indicate standard deviations.

**Figure 2:**
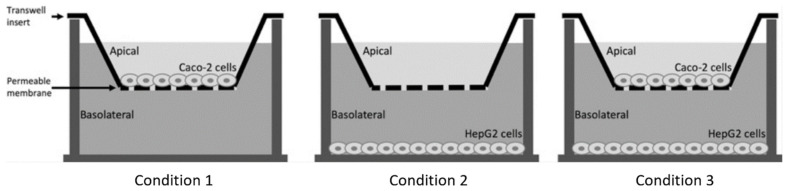
Schematic overview of different experimental conditions were performed in the transwell system.

Caco-2 cells and HepG2 cells were both cultured in T75 flasks (Corning, Cambridge, MA, USA) at 37 °C in a humidified atmosphere of 5% carbon dioxide (CO_2_) in medium (DMEM for Caco-2 and MEM for HepG2). Cell media contained 10% heat inactivated FBS, 1% sodium pyruvate, 1% NEAA and 1% penicillin-streptomycin mixture. For all experiments, Caco-2 cells were seeded to the inside of transwell inserts (1.5 mL/well) at a density of 300,000 cells/mL. For the first 21 days, the inserts were placed in 6-well plates. During these 21 days, the Caco-2 cells were allowed to differentiate to their small intestinal phenotype [19]. Next, the inserts were placed in new 6-well plates to determine whether the Caco-2 cell layers were confluent by examining phenol red leakage from the apical to the basolateral compartment [20]. For this, MEM with phenol red was added to the apical compartment, while MEM without phenol red was added to the basolateral compartment for 48 h at 37 °C in a humidified atmosphere of 5% CO_2_. The optical density (OD) of the medium solution in both compartments was measured at 450 nm using a spectrophotometer (Beckman, Pasadena, CA, USA) (Figure 1). Only confluent Caco-2 monolayers (OD 450 nm of the basolateral medium less than 0.02) were used in the experiments. In addition, HepG2 cells were seeded in 6-well plates (2.6 mL/well), at a density of 300,000 cells/mL, and cultured for 48 h. Three different conditions were used (Figure 2). First, Caco-2 cells were seeded in the inserts, without HepG2 cells in the lower compartment. Second, HepG2 cells were seeded in the lower compartment without Caco-2 cells on the insert above. Finally, Caco-2 cells were seeded on the insert in addition to HepG-2 cells in the lower compartment, and both of them were incubated together in a co-culture system to mimic the intestine–liver interaction. The three different setups of the transwell system were allowed to equilibrate for 48 h before the start of the experiments.

Several experiments were performed. In condition 1, C4 was added to the apical surface of Caco-2 cells in a concentration range of 0, 1, 2, 4, and 6 mM. In condition 2, C4 was added in the same concentration range to HepG2 cells. In condition 3, C4 was added to the apical surface of Caco-2 cells, again in the same concentration range, but this time HepG2 cells were present in the basolateral compartment. In this co-culture condition, we also evaluated the effect of an inflammatory component. For this, a cytokine cocktail of TNFα (with final concentration of 100 ng/mL) and IL-1β (with final concentration of 5 ng/mL) was added either only apically to the Caco-2 cells, or only to HepG2 cells in the basolateral compartment, or simultaneously to both the apical and basolateral compartments. JQ1(+), a BET inhibitor, was included as a positive control in a separate well to ensure that cells were responsive to the treatments and produced sufficient amounts of ApoA-I mRNA [21]. For this, JQ1(+) was added in final concentrations of 3 µM. Both C4 and JQ1(+) were dissolved in dimethyl sulfoxide (DMSO, cell culture tested), and effects were expressed relative to those of the carrier control (DMSO only). The final DMSO concentration was 0.2% in all samples. In all experiments, both Caco-2 and HepG2 cells were harvested for analysis of ApoA-I mRNA expression after lysing with Tri-reagent, as described [21] and stored at −80 °C until further analysis.

### 2.3. Quantification of Gene mRNA Transcription

To evaluate the effects of C4 on ApoA-I mRNA expression, total RNA was isolated from the Caco-2 and HepG2 cells using Tri-reagent and the RNeasy mini kit (Qiagen, Hilden, Germany) according the manufacturer’s instructions. For cDNA synthesis, 350 ng of total RNA was reverse transcribed using moloney murine leukemia virus (MMLV) reverse trans, dNTPs, random hexamers, dithiothreitol (DTT) and a 5xFS buffer supplemented with RNAse inhibitor (Thermo Fisher Scientific, Bleiswijk, The Netherlands). The resulting cDNA was used for real time quantitative PCR using TaqMan Gene Expression Assays using Cyclophilin A (Hs99999904) as a housekeeping control. To quantify ApoA-I, the TaqMan Gene Expression Assays (Hs00163641) was used. Values are presented as relative gene expressions based on Ct values, normalized for the internal control Cyclophilin A and compared with control conditions.

### 2.4. Statistical Analysis

All independent dose-response experiments with C4 were performed in duplicate, and each experiment was repeated three times. Six biological (12 technical) replicates were performed for every single C4 dose. Regression analysis was used to examine dose-response relationships between the concentration of added C4 and the mRNA expression of ApoA-I. For a dose-response relationship, the regression coefficients had to be significantly different from zero at (*p* < 0.05). Effects of the positive control JQ1(+) in Caco-2 or HepG2 cells when cultured alone were statistically evaluated versus the control condition determined by a Mann-Whitney U test, in which a *p*-value < 0.05 was considered statistically significant. When evaluating the effects of JQ1(+) on ApoA-I mRNA expression in the different pro-inflammatory cytokines conditions in HepG2 or Caco-2 cells, the Mann-Whitney U test was used with a correction for multiple comparisons, in which a *p*-value < 0.008 was considered statistically significant. In addition, when evaluating the effects of adding no cytokines versus cytokines added apical, basolateral, or to both compartments side-by-side, the Mann-Whitney U test was also used with a correction for multiple comparisons (requiring *p* < 0.008 for significance). All statistical analyses were performed using SPSS v.25 (IBM Corp., Armonk, NY, USA).

## 3. Results

### 3.1. Effects of C4 on ApoA-I mRNA Expression in Caco-2 or HepG2 Cells (Conditions 1 and 2)

**Figure 3:**
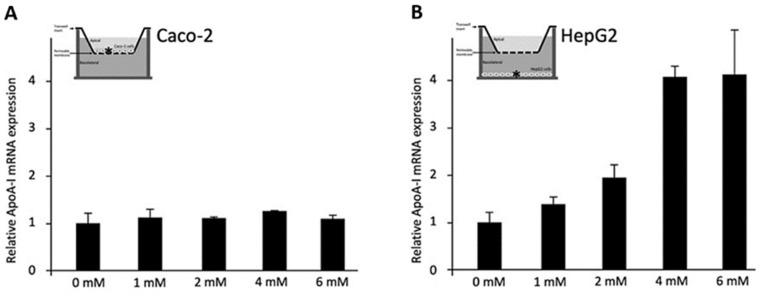
Relative Apolipoprotein A-I (ApoA-I) mRNA expressions in Caco-2 and HepG2 cells treated with different concentrations of C4. (**A**) Increasing C4 concentrations did not have any significant effects on ApoA-I mRNA expression in Caco-2 cells. (**B**) Increasing C4 concentrations showed a significant increase in ApoA-I mRNA expression in HepG2 cells (*p* < 0.001). All results are presented as the mean, while error bars indicate standard deviations. Data were normalized against the expression observed in the control condition, which was arbitrarily set at 1. A linear regression was performed for C4 dose-response effects. Changes were considered significant when the regression coefficients were significantly different from zero (*p* < 0.05). ApoA-I, apolipoprotein-I; mRNA, messenger RNA.

To evaluate the effects of C4 on Caco-2 cells (Condition 1, Figure 3A), different doses of C4 were added to the apical surface of the Caco-2 cells on the insert, without HepG2 cells in the lower compartment. C4 did not change ApoA-I mRNA expression in the Caco-2 cells. When C4 was added to HepG2 cells in the lower compartment without the Caco-2 cells on the insert above (Condition 2, Figure 3B), ApoA-I mRNA expression dose-dependently increased (*p*  <  0.001), with a maximum of a 4.2-fold at a 6 mM concentration of C4.

### 3.2. Effects of C4 Added to the Apical Surface of Caco-2 Cells on ApoA-I mRNA Expression in Caco-2 and HepG2 Cells (Condition 3)

**Figure 4:**
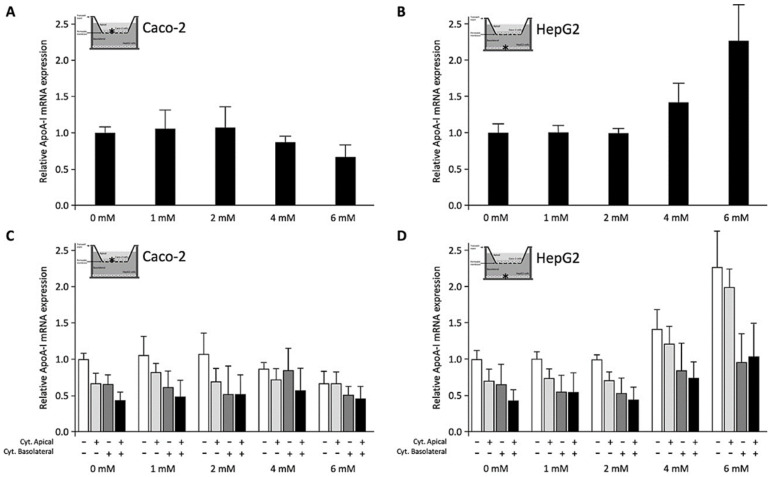
Relative Apolipoprotein A-I (ApoA-I) mRNA expressions in Caco-2 and HepG2 cells were cultured in a transwell system and treated with different concentrations of C4. All results are presented as the mean, while error bars indicate standard deviations. Data were normalized against the expression observed in the control condition, which was arbitrarily set at 1. A linear regression was performed for C4 dose–response effects. Changes were considered significant when the regression coefficients were significantly different from zero (*p* < 0.05; **A**–**D**). When evaluating the effects of adding no cytokines versus cytokines added apical, basolateral or to both compartments side-by-side, the Mann-Whitney U test was used with a correction for multiple comparisons in which a *p*-value < 0.008 was considered statistically significant (**C**,**D**). (**A**) Increasing C4 concentrations showed a significant reduction in ApoA-I mRNA expression in Caco-2 cells that were cultured alone (*p* < 0.01). (**B**) Increasing C4 concentrations showed a significant increase in ApoA-I mRNA expression in HepG2 cells that were cultured alone (*p* < 0.001). (**C**) Increasing C4 concentrations did not show any significant effects on ApoA-I mRNA expression in Caco-2 cells co-cultured with HepG2 cells in the presence of cytokines (either apical, basolateral or both compartments). (**D**) Increasing C4 concentrations showed a significant increase in ApoA-I mRNA expression in HepG2 cells co-cultured with Caco-2 cells in the presence of cytokines (either apical, basolateral or both compartments) (*p* < 0.01). When cytokines were added to the apical side of the Caco-2 cells, ApoA-I mRNA expression significantly (*p* <  0.008) decreased in both Caco-2 and HepG2 cells (**C**,**D**). A significant reduction in ApoA-I mRNA expression (*p*  <  0.008) was observed in Caco-2 and HepG2 cells when cytokines were added to the basolateral compartment instead of the apical side. When the cytokines were added to both the apical and basolateral compartments, there was an even further reduction (*p*  <  0.008) in ApoA-I mRNA expression in Caco-2 and HepG2 cells. The presence of cytokines was indicated with (+), while the absence of cytokines was indicated with (−). ApoA-I, apolipoprotein-I; mRNA, messenger RNA.

To explore the effects of C4 on Caco-2 and HepG2 cells co-cultured in a transwell system, C4 was added to the apical surface of Caco-2 cells cultured on inserts placed on top of the HepG2 cells in the lower compartment (Condition 3). In contrast to the condition without HepG2 cells (Figure 3A), ApoA-I gene expression in Caco-2 cells co-cultured with HepG2 cells significantly (*p* < 0.01) decreased after adding C4 (Figure 4A), whereas at the same time, ApoA-I mRNA expression in HepG2 cells was dose-dependently increased (*p*  <  0.001) (Figure 4B).

When cytokines were added to the apical side of the Caco-2 cells, ApoA-I mRNA concentrations were significantly (*p*  <  0.008) lowered in both Caco-2 and HepG2 cells. Also, a significant decrease (*p*  <  0.008) was observed in both cell lines when cytokines were added to the basolateral compartment instead of the apical side. When cytokines were added to the apical side and basolateral compartment simultaneously, there was even a further reduction (*p*  <  0.008) in ApoA-I mRNA in both cell lines (Figure 4C,D). When C4 was added apically to the Caco-2 cells in the presence of cytokines (either apical, basolateral or both), C4 did not change ApoA-I mRNA expression in the Caco-2 cells (Figure 4C). In HepG2 cells, ApoA-I mRNA expression dose-dependently increased after adding C4 apically to the Caco-2 cells (*p*  <  0.01), also in the presence of cytokines either added apical, basolateral or in both compartments (Figure 4D). In other words, apical C4 was able to rescue the reduced ApoA-I mRNA expression in HepG2 cells exposed to pro-inflammatory cytokines but not in intestinal Caco-2 cells.

### 3.3. Effects of the Positive Control JQ1(+) on ApoA-I mRNA Expression in Caco-2 and HepG2 Cells

**Figure 5:**
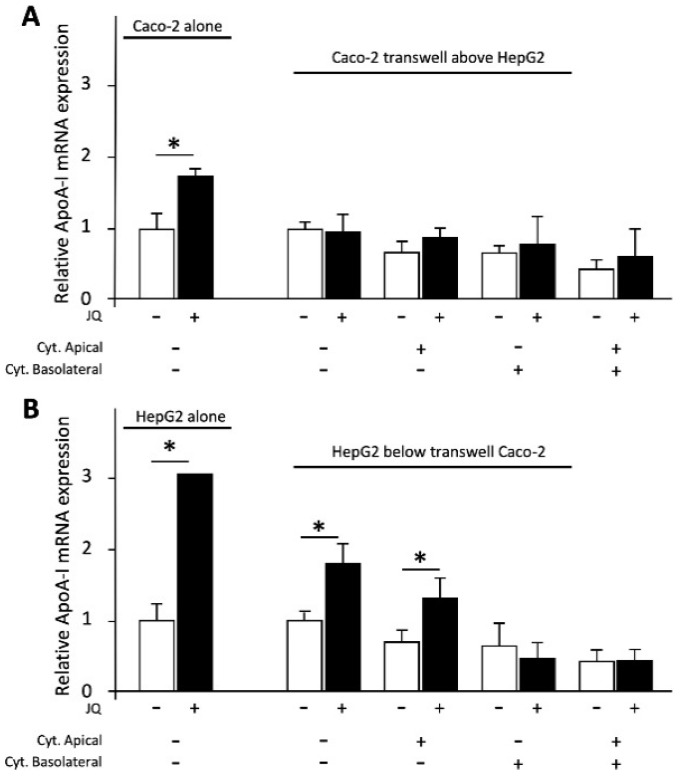
Relative Apolipoprotein A-I (ApoA-I) mRNA expressions in Caco-2 and HepG2 cells were cultured in a transwell system and treated with the positive control JQ1(+) (3 μM). (**A**) JQ1(+) significantly increased ApoA-I mRNA expression in Caco-2 cells that were cultured alone (*p* < 0.05). JQ1(+) did not affect ApoA-I gene expression in Caco-2 cells co-cultured with HepG2 cells without cytokines or when cytokines were added to either apical, basolateral or both compartments. (**B**) JQ1(+) significantly increased ApoA-I mRNA expression in HepG2 cells that were cultured alone (*p* < 0.01). JQ1(+) significantly increased ApoA-I mRNA expression in HepG2 cells co-cultured with Caco-2 cells without cytokines or when cytokines were added to apical compartments (*p* < 0.008). JQ1(+) did not affect ApoA-I gene expression in HepG2 cells co-cultured with Caco-2 cells when cytokines were added basolaterally or to both compartments. All results are presented as the mean, while error bars indicate standard deviations. Data were normalized against the expression observed in the control condition, which was arbitrarily set at 1. A Mann-Whitney U test was performed to evaluate JQ1(+) effects in Caco-2 or HepG2 cells that were cultured alone versus control conditions, in which a *p*-value < 0.05 was considered statistically significant. When evaluating the effects of JQ1(+) on ApoA-I mRNA expression in different pro-inflammatory cytokines conditions in HepG2 or Caco-2 cells, the Mann-Whitney U test was used with a correction for multiple comparisons, in which a *p*-value < 0.008 was considered statistically significant. Changes are indicated with * when the effect of JQ1(+) is significantly different from the control. The presence of cytokines was indicated with (+), while the absence of cytokines was indicated with (−). ApoA-I, apolipoprotein-I; mRNA, messenger RNA.

The positive control JQ1(+) increased ApoA-I mRNA expression (*p* < 0.05) in Caco-2 or HepG2 cells when cultured alone (Figure 5). As mentioned before and shown in Figure 4C,D, ApoA-I mRNA expression in cells exposed to pro-inflammatory cytokines was lower compared with mRNA expression in non-inflamed cells. Effects of JQ1(+) treatment in these inflammatory conditions are shown in Figure 5. JQ1(+) did not affect ApoA-I gene expression in Caco-2 cells (co-cultured with HepG2 cells) without cytokines or when cytokines were added to either apical, basolateral or both compartments. Moreover, JQ1(+) significantly (*p* < 0.008) increased ApoA-I gene expression in HepG2 cells (co-cultured with Caco-2 cells) with or without cytokines added to the apical compartment. Furthermore, JQ1(+) did not affect ApoA-I gene expression in HepG2 cells (co-cultured with Caco-2 cells) with cytokines added basolaterally or to both compartments (Figure 5). Taken together, the cells responded to JQ1(+) as expected [21].

## 4. Discussion

In earlier experiments, we have shown that C4 treatment increased ApoA-I mRNA expression in HepG2 cells in both normal and inflammatory conditions [9,12]. These positive effects on hepatic ApoA-I transcription were observed when C4 was added directly to HepG2 cells. Here, we have evaluated whether these effects were still evident when C4 was added to the apical surface of enterocytes, which means that it first had to be taken up by Caco2 cells and transported towards HepG2 cells in the basolateral compartment. This also made it possible to evaluate the effects of C4 on ApoA-I expression in Caco2 cells, which also contribute to ApoA-I concentrations in the circulation [22]. Although C4 is mainly produced by microbiome in the colon, we decided to examine the effects of C4 on ApoA-I transcription in small intestinal enterocytes since ApoA-I is mainly produced in the duodenum and jejunum [23,24]. However, although SCFAs as end products of bacterial fermentation are present in higher concentrations in the colon, SCFAs are also present in the proximal intestines [23,25]. We did not find any effect of C4 on ApoA-I gene expression in Caco2 cells when cultured alone (condition 1). In contrast to the effects in Caco-2 cells, but in line with our earlier findings [9], C4 again significantly increased ApoA-I expression in HepG2 cells (condition 2). Interestingly, in the co-culture model (condition 3), we found that ApoA-I mRNA expression in Caco-2 cells was even lowered after adding C4 in the presence of HepG2 cells, whereas effects of C4 on HepG2 cells remained positive, showing a significant increase in ApoA-I gene expression. This illustrates the cell-specific response of enterocytes and hepatocytes in a co-culture model in response to C4 exposure. Moreover, in the presence of cytokines either added to apical, basolateral or to both cell compartments, ApoA-I mRNA levels in HepG2 cells were rescued by C4. However, the effects of C4 on elevating ApoA-I mRNA expression in HepG2 cells when added to the apical side of Caco-2 cells in the transwell were lower compared with the effects of C4 when added directly to HepG2 cells. The question is how the lower hepatic effects of C4 in the co-culture experiments can be explained. Theoretically, decreased bioavailability (i.e., the amount of SCFAs that are transported from the apical to the basolateral side), which translates into lower hepatic exposure to C4, seems the most logical explanation. It is well-known that SCFAs are used as fuel by the intestinal cells [26]. Therefore, once SCFAs are metabolized by the intestinal cells, they will not be available (in their original concentrations) in the basolateral compartment, hence explaining the lower bioavailability. Moreover, we also observed that the inhibitory effect on hepatic ApoA-I expression was larger when cytokines were added directly to HepG2 cells. This finding, in the case of cytokines, could also attributed to the bioavailability of cytokines. However, in contrast to C4, cytokines are not used for fueling enterocytes, which again raises the question of why cytokines’ effects on HepG2 cells were smaller when they were added apically in the transwell system. An alternative explanation for the lower effects of both C4 and cytokines on HepG2 cells after adding them to Caco-2 cells could also theoretically be a cross-talk phenomenon between Caco-2 cells and HepG2 cells. For example, the secreted factors from the Caco-2 cells into the basolateral compartment might influence the effects of C4 on HepG2 cells. In theory, such cross-talk between intestine and liver may be critical for human health since several liver diseases result from alterations in the intestinal barrier [27]. Moreover, such interactions between the intestine, including its microbiota, and the liver can also be regulated by exposure to dietary compounds [27]. A recent study investigated the effects of SCFAs on the gut-liver axis in interconnected human micro-physiological systems (MPS) [28]. Adding SCFAs to the apical enterocyte side favorably modulated the gut-liver axis in MPS by innate immune inactivation. Moreover, Trapecar et al. also showed, by using the same MSP system, that apical addition of SCFAs to enterocytes not only inhibited gut inflammation but also increased liver metabolic function, improved lipid metabolism and enhanced hepatic bile acid secretion [28]. Altogether, this illustrates that hepatic effects exist after intestinal exposure to SCFAs. However, this can still be simple response to SCFAs transferred from the intestine to the liver, or cross-talk of the liver responding to factors secreted via intestinal cells. To the best of our knowledge, there is so far no known intestinal factor that is secreted in response to SCFAs exposure, influencing hepatic physiology. However, our data may suggest the existence of such a factor. We showed that Caco-2 cells responded in the opposite direction to C4 compared with HepG2 cells. To elaborate, treating Caco-2 cells with C4 had no effects on ApoA-I mRNA when the Caco-2 cells were cultured alone and even decreased intestinal ApoA-I mRNA expression in the co-culture model. The question now is whether we should expect effects on the intestine and liver to go hand-in-hand or whether there are other known conditions where effects on the liver and intestine were also in the opposite direction. A potential difference in ApoA-I mRNA response between the intestinal and hepatic cells could be explained by the need of some coactivators to produce ApoA-I, which is absent in Caco-2 cells. For example, the synergy between the ApoA-I promoter and the ApoCIII enhancer is essential to induce intestinal ApoA-I transcription, whereas the induction of hepatic ApoA-I gene expression seems independent of the ApoCIII enhancer [29]. However, although the absence of such coactivators might explain why C4 had no effects on Caco-2 cells when cultured alone, we have now even observed inhibition of ApoA-I expression in Caco-2 in the co-culture model after C4 exposure. This suggests that effects of C4 on Caco-2 cells might activate different pathways that not only translate into lower ApoA-I transcription but could also induce the production of a currently unknown factor. These factors might not only affect ApoA-I transcription in the enterocyte itself but also influence hepatic function, which is evident in our studies as a lower hepatic ApoA-I transcription. Identification of such a factor would be highly informative and deserves further studies. Finally, although we acknowledge that the various SCFAs have different systemic concentrations, we expect other SCFAs to behave identically [1]. On the other hand, as explained, it could be that there are differences regarding intestinal cells metabolism between different SCFAs, which might consequently result in different basolateral concentrations and different effects on liver cells. However, since the concentrations of acetate and propionate in vivo are likely to be higher than those of butyrate, it seems “safe” to speculate that comparable effects will be observed for other SCFAs.

In conclusion, our findings indicate that in co-culture experiments, adding C4 apically to intestinal cells does not increase the lower ApoA-I mRNA level in inflamed Caco-2 cells. Furthermore, C4 added apically to Caco-2 cells elevates hepatic ApoA-I transcription and rescues the lower ApoA-I expression in inflamed HepG2 cells. As we found previously that all SCFAs (in line with C4) were able to enhance hepatic ApoA-I expression when directly added to HepG2 cells [9], we suggest further exploration of the effects of other SCFAs in the intestine-liver co-culture model. Moreover, the effects of adding C4 to the apical side of enterocytes translate into a smaller effect on HepG2 cells compared with adding C4 to hepatocytes directly. We speculate that this could be due to lower bioavailability, but it could also indicate cross-talk between intestine and liver. Identification of such an enterocyte-derived inhibitory factor warrants further studies.

## Data Availability

The data that support the findings of this study are available from the corresponding author, upon reasonable request.

## Abbreviations

ApoA-I	Apolipoprotein A-I
cDNA	Complementary DNA
IL-1β	Interleukin 1 beta
NF-κB	Nuclear factor kappa B
SCFAs	Short chain fatty acids
TNFα	Tumor necrosis factor-α
mRNA	messenger RNA
